# The Relationship between Vitamin D and Basal Cell Carcinoma: A Systematic Review

**DOI:** 10.7759/cureus.29496

**Published:** 2022-09-23

**Authors:** Rana Abdelwahab, Ruimin Huang, Shanthi Potla, Sushen Bhalla, Yousif AlQabandi, Savitri Aninditha Nandula, Chinmayi Sree Boddepalli, Sai Dheeraj Gutlapalli, Vamsi Krishna Lavu, Lubna Mohammed

**Affiliations:** 1 Dermatology, California Institute of Behavioral Neurosciences & Psychology, Fairfield, USA; 2 Dermatology, Mansoura University, Mansoura, EGY; 3 Internal Medicine, California Institute of Behavioral Neurosciences & Psychology, Fairfield, USA; 4 Psychiatry and Behavioral Sciences, California Institute of Behavioral Neurosciences & Psychology, Fairfield, USA; 5 Medical College, Avalon University School of Medicine, Cleveland, USA; 6 Ministry of Health, Al Bahar Ophthalmology Center, Sabah Area, KWT

**Keywords:** 25 dhd vitamin, ergocalciferol 25 hydroxyvitamin d2, hydro d3 cholecalciferol, non-melanoma skin cancer, basal cell carcinoma, cholecalciferol, vitamin d

## Abstract

This systematic review studies the relationship between vitamin D serum levels and basal cell carcinoma (BCC). The primary source of vitamin D is sunlight exposure. Recently, an increase in the intake of vitamin D supplements has been noticed. The protective value of vitamin D is well established and has been studied several times for the health of the bones, cartilage, growth, various dermatological diseases, and also as a chemoprotective agent against several cancers. On the scientific front, it has yet to be established that increasing serum vitamin D levels increase the incidence of BCC. We included reports that investigated this relationship in this review. We applied keywords in published papers in PubMed, ScienceDirect, Cochrane, and Google Scholar to find relevant studies. After applying the Preferred Reporting Items for Systematic Reviews and Meta-Analysis (PRISMA) checklist and the quality appraisal for 68 records, we included only ten studies. In these studies, serum levels of vitamin D were measured. Five of them supported the link between BCC incidence and development and high serum vitamin D levels (e.g., Mahamat-Saleh Y, et al.), while the other five did not (e.g., Tang JY, et al.). We included only two studies that investigated the vitamin D receptor (VDR) polymorphism. Experts debate adding a high dose of vitamin D supplements to our daily routine. After studying most of the reports, it was ascertained that the literature supports keeping vitamin D serum levels below 30-60 nmol/L. However, further studies should be done to help find a healthy balance of vitamin D serum levels, especially when it comes to increasing the risk of cancer like BCC.

## Introduction and background

Skin cancer is the most common malignancy among Caucasians, with basal cell carcinoma (BCC) being the commonest among them in 2012 [1,2]. Its prevalence in the United States has risen to 800 to 1000 cases per 100,000 people [1,3,4]. With a dramatic increase in the incidence of both melanoma and non-melanoma skin cancers over the past 50 years, more than one million new diagnoses of BCC appear each year [5]. According to the World Health Organization (WHO) in 2006, light skin types (Fitzpatrick I, II) are more prone to developing keratinocyte-derived cancer as they are predisposed to sunburns after exposure to ultraviolet rays (UVR) [2].

Both natural and artificial ultraviolet (UV) radiation, particularly ultraviolet-B (UV-B) with a wavelength range between 280 and 320 nm, is the most important risk factor for BCC, especially for light skin types (Fitzpatrick I, II) [2,3]. Modifiable risks that increase UV exposure include wearing sleeveless or light clothing, participating in outdoor activities, taking suntan baths, and not applying sunscreen. Other non-modifiable risk factors are increased life expectancy, ozone depletion [6], genetic alterations, and UV radiation used for treating some skin conditions like psoriasis and vitiligo [2,6,7]. Sunlight exposure has a dual effect; it helps our skin produce vitamin D, exerting a protective effect against the development of skin cancer as well as causing deoxyribonucleic acid (DNA) damage to our skin cells [3]. 

Vitamin D is a fat-soluble vitamin that has two natural forms: ergocalciferol (vitamin D2) and cholecalciferol (vitamin D3). We get them by consuming fish, dairy products, and cereal products, as well as fortified foods or by taking dietary supplements. This accounts for almost 20% of our total vitamin D requirements [8,9,10]. Most vitamin D, 80%, is synthesized by exposure to ultraviolet radiation (UVR) in the skin, where 7-dehydrocholesterol (7-DHC) is converted into vitamin D3. Vitamins D2 and D3 are then metabolized in the liver to produce 25-hydroxy vitamin D (25(OH)D), the major circulating form that reflects vitamin D status [5]. 

Vitamin D is involved in various biologic functions, including bone health, antiproliferative, anti-angiogenic, and modulation of the immune system. It has been studied for its protective role against several types of cancer [8-11]. However, recent epidemiologic studies have addressed the association between vitamin D and skin cancer risk [5,6,12-7]. They investigated circulating levels of 25-hydroxy vitamin D (25(OH)D), a biomarker of vitamin D status that reflects both intake and synthesis in response to sun exposure [18,19]. High 25(OH)D levels were associated with an increased risk of melanoma and keratinocyte cancers (KC) in most studies [6,13], but some studies found inverse or null associations [4]. While a recent meta-analysis of four studies found no link between high serum 25(OH)D levels and melanoma risk, high 25(OH)D levels have been linked to an increased risk of keratinocyte carcinoma (KC), particularly BCC [5,16]. 

Photochemical changes in the skin are caused by UV-B radiation, which leads to skin cancer [3,8]. There has been extra interest in the last few years regarding polymorphism, in particular genes whose products are induced by UVR, which may increase our risk of developing skin cancer. These genes encode the vitamin D receptor (VDR) and methylenetetrahydrofolate reductase (MTHFR) [6,7,8,18,19]. The VDR is a member of the transacting transcriptional regulatory factor superfamily that includes steroid and thyroid hormone receptors, as well as retinoid-X receptors and retinoic acid receptors [3], and it is expressed on a variety of cells, including epidermal basal keratinocytes [2,19,20]. 1,25-Dihydroxy vitamin D3 (1,25(OH)2D3) exerts its action by binding to VDR, which then translocates to the nucleus where it regulates the expression of VDR-responsive genes. Some of these genes play a role in the induction of cell differentiation and suppression of proliferation [19]. The VDR gene is highly polymorphic as it has been found in more than six hundred and eighteen variants. Most of the polymorphisms that have been studied in association with skin cancer risk are FokI (rs2228570), ApaI (rs7975232), BsmI (rs1544410), and TaqI (rs731236) [7,19].

We are interested in studying the effect of circulating 25(OH)D levels in the blood on the development of primary or recurrent basal cell carcinoma (BCC) in this systematic review. Since most of the population, especially the elderly, pay extra attention to their bone health, as well as for specific cancer prevention and seek a better quality of life, they are directed toward adding extra over-the-counter (OTC) supplements and increasing their nutrients without paying attention to the downsides of extra intake. Is it possible that the increased intake of vitamin D supplements has increased the incidence of BCC worldwide? This systematic review aims to explore the relationship between vitamin D serum levels and the risk of developing or preventing BCC. As a known sequence, UV radiation leads to VDR polymorphism, which causes BCC. What we would like to explore is whether the increase of vitamin D, due to UV radiation and the intake of the supplement, causes BCC or whether the decrease of vitamin D due to VDR polymorphism causes BCC.

Methods

**Figure 1:**
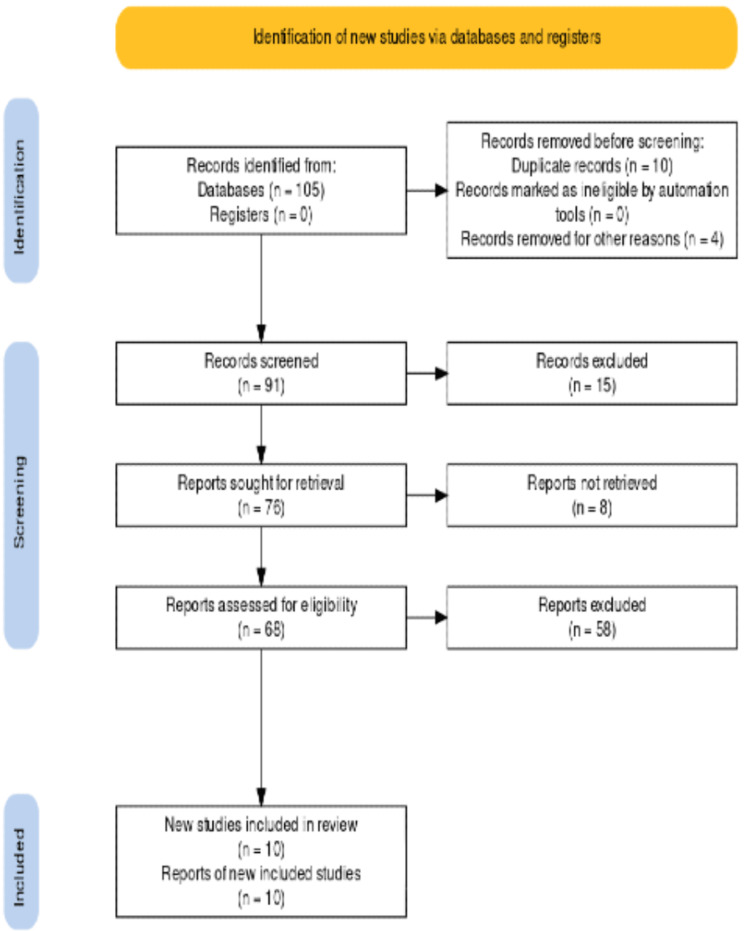
The Preferred Reporting Items for Systematic Reviews and Meta-Analysis (PRISMA) 2020 guidelines

The Preferred Reporting Items for Systematic Reviews and Meta-Analysis (PRISMA) 2020 guidelines were followed in this systematic review [21], as shown in Figure 1. The population, intervention, comparison, and outcome (PICO) formats were incorporated into this study design.

Databases and search strategy

Detailed research was conducted using the keywords mentioned in Table 1 to recognize the studies analyzing and assessing the effect of vitamin D on both the occurrence and progression of BCC using PubMed as the primary database, and other studies were collected from Google scholar, Cochrane Library, and ScienceDirect as well.

These keywords were based on the keywords used in previous literature and through Medical Subject Headings (MeSH) depending on the database used. All the articles considered were chosen without restriction to the study type, i.e., traditional reviews, systematic reviews, clinical trials, case-control, and cohort studies. Studies were collected starting from 2010 to 2022; they were not refined based on age, ethnicity, or demographical limitations. All the articles chosen were in the English language.

**Table 1:** Keywords used in the systematic review

PubMed	Vitamin D: Cholecalciferol OR Hydro D3 cholecalciferol OR Ergocalciferols O25 Hydroxyvitamin D 2 OR 1,25dhd vitamin AND Basal cell carcinoma: Basal cell cancer OR Non-melanoma skin cancer	("Calcitriol/administration and dosage"[Mesh] OR "Calcitriol/adverse effects"[Mesh] OR "Calcitriol/analogs and derivatives"[Mesh] OR "Calcitriol/biosynthesis"[Mesh] OR "Calcitriol/blood"[Mesh] OR "Calcitriol/classification"[Mesh] OR "Calcitriol/etiology"[Mesh] OR "Calcitriol/history"[Mesh] OR "Calcitriol/metabolism"[Mesh] OR "Calcitriol/organization and administration"[Mesh] OR "Calcitriol/pharmacokinetics"[Mesh] OR "Calcitriol/physiology"[Mesh] OR "Calcitriol/poisoning"[Mesh] OR "Calcitriol/radiation effects"[Mesh] OR "Calcitriol/standards"[Mesh] OR "Calcitriol/therapeutic use"[Mesh] OR "Calcitriol/toxicity"[Mesh] AND ( "Carcinoma, Basal Cell/anatomy and histology"[Mesh] OR "Carcinoma, Basal Cell/blood"[Mesh] OR "Carcinoma, Basal Cell/blood supply"[Mesh] OR "Carcinoma, Basal Cell/chemically induced"[Mesh] OR "Carcinoma, Basal Cell/classification"[Mesh] OR "Carcinoma, Basal Cell/complications"[Mesh] OR "Carcinoma, Basal Cell/cytology"[Mesh] OR "Carcinoma, Basal Cell/diagnosis"[Mesh] OR "Carcinoma, Basal Cell/diagnostic imaging"[Mesh] OR "Carcinoma, Basal Cell/diet therapy"[Mesh] OR "Carcinoma, Basal Cell/drug therapy"[Mesh] OR "Carcinoma, Basal Cell/economics"[Mesh] OR "Carcinoma, Basal Cell/epidemiology"[Mesh] OR "Carcinoma, Basal Cell/etiology"[Mesh] OR "Carcinoma, Basal Cell/genetics"[Mesh] OR "Carcinoma, Basal Cell/history"[Mesh] OR "Carcinoma, Basal Cell/metabolism"[Mesh] OR "Carcinoma, Basal Cell/microbiology"[Mesh] OR "Carcinoma, Basal Cell/mortality"[Mesh] OR "Carcinoma, Basal Cell/organization and administration"[Mesh] OR "Carcinoma, Basal Cell/pathology"[Mesh] OR "Carcinoma, Basal Cell/physiology"[Mesh] OR "Carcinoma, Basal Cell/physiopathology"[Mesh] OR "Carcinoma, Basal Cell/prevention and control"[Mesh] OR "Carcinoma, Basal Cell/radiotherapy"[Mesh] OR "Carcinoma, Basal Cell/secondary"[Mesh] OR "Carcinoma, Basal Cell/statistics and numerical data"[Mesh] OR "Carcinoma, Basal Cell/ultrastructure"[Mesh] )
Google Scholar	Vitamin D AND Basal cell carcinoma	
Cochrane Library	Basal cell carcinoma AND Vitamin D	
ScienceDirect	Skin Basal cell carcinoma AND Vitamin D	

All references were grouped using Endnote for duplicate removal. The records were initially reviewed by two independent scientists based on the titles and abstracts, and the irrelevant studies were excluded. After reviewing with the group, a retrieval of the full-text articles was done. Because of the few systematic reviews, meta-analyses, and narrative reviews on this topic, the investigators decided not to exclude them from the study.

Eligibility criteria 

The studies were selected based on the Participants, Intervention, Comparison, and Outcomes (PICO) elements.

Participants: basal cell carcinoma patients and participants on vitamin D supplements. Intervention: serum vitamin D levels were measured. Comparison: Two or more groups were compared. Outcomes: basal cell carcinoma

Additional inclusion and exclusion criteria were added.

Inclusion criteria: English-language, free full-text articles published since 2010; systematic reviews, meta-analyses, randomized controlled trials, and observational and clinical trials were included. Both women and men of all ages were included.

Exclusion criteria: ecological studies and editorials were excluded from the systematic review. Studies that focused on the relationship between vitamin D exposure and survival from melanoma, recurrence, or prognostic factors for melanoma (e.g., tumor thickness, ulceration) were also excluded.

Results 

Study Selection and Quality Assessment 

We identified 105 potentially eligible records across all the databases. We used Endnote to remove the duplicates, which were 10 papers, and excluded four more. Ninety-one were screened, and we removed 15 of them because they were not relevant to our study. Another eight were removed as well because they didn't meet our inclusion and exclusion criteria. Sixty-eight were assessed for eligibility. We excluded the following: 15 studies because of the high risk of bias; 20 studies because the target outcome was not found; and 23 studies that did not have our target patients. Finally, each publication was given a quality rating, and 10 studies with a score of higher than 70% were admitted into the review (Figure 1). The quality of the final papers selected was also verified by a second author to decrease the risk of bias. There were no more resources uploaded.

Risk of Bias in Individual Studies 

The full articles remaining were assessed for quality assessment and risk of bias using tools depending on the study type: Randomized Control Trials (RCTs), Cochrane Collaboration Risk of Bias Tool (CCRBT); cohort studies, Newcastle Ottawa Scale (NOS); case-control studies, Joanna Briggs Institute (JBI) Critical Appraisal Checklist; and systematic reviews and meta-analyses, Assessment of Multiple Systematic Reviews 2 (AMSTAR 2) [16-20]. Each assessment tool had its own criteria and different scoring. A point was given when a tool scored "LOW RISK," "YES," "PARTIAL YES," or "1". Two points were given when "2" was indicated. A score of at least 70% for each assessment tool was accepted (Table 2). 

**Table 2:** Quality assessment of each type of study. JBI - Joanna Briggs Institute, CCRBT - Cochrane Collaboration Risk of Bias Tool, NOS - Newcastle Ottawa Scale, AMSTAR 2 - Assessment of Multiple Systematic Reviews 2, RCTs - randomized controlled trials, RoB - risk of bias

Quality assessment tool	Type of study	Items & their characteristics	Total score	Accepted score (>70%)	Accepted studies
JBI [22]	Case-Control	10 points: (1) Were the groups comparable other than the presence or absence of disease in cases and controls? (2) Were the cases and controls matched appropriately? (3) Were the same criteria used for the identification of cases and controls? (4) Was exposure measured in a standard, valid, and reliable way? (5) Was exposure measured in the same way for cases and controls? (6) Were confounding factors identified? (7) Were strategies to deal with confounding factors stated? (8) Were outcomes for cases and controls assessed in a consistent, valid, and reliable manner? (9) Was the exposure period of interest long enough to be meaningful? (10) Was appropriate statistical analysis done? Answers: Yes, No, Unclear or Not/Applicable	9	7	Vornicescu C et al. (2), Ince B et al. (6), Lesiak A et al. (19)
CCRBT [23]	RCTs	Seven points: random sequence generation and allocation concealment (selection bias), selective outcome reporting (reporting bias), other sources of bias, blinding of participants and personnel (performance bias), blinding of outcome assessment (detection bias), and incomplete outcome data (attrition bias). Bias assessed as LOW RISK, HIGH RISK, or UNCLEAR.	7	5	Tang JY et al. (4)
NOS [24]	Cohort	Eight points: (1) Representativeness of the exposed cohort (2) Selection of the non-exposed cohort (3) Ascertainment of exposure (4) Demonstration that the outcome of interest was not present at the start of the study (5) Comparability of cohorts on the basis of the design or analysis* (6). Assessment of the outcome (7) Was the follow-up long enough for outcomes to occur? (8) Adequacy of cohort follow-up Scoring was done by placing a point on each category. Scored as 0, 1, 2. * Maximum of two points are allotted in this category.	8	6	Park SM et al. (12), Eide MJ et al. (14), Liang G et al. (15), Tang JY et al. (16)
AMSTAR 2 [25]	Systematic review, Meta-analysis	16 points: (1) Did the research questions and inclusion criteria for the review include the components of PICO? (2) Did the report of the review contain an explicit statement that the review methods were established prior to the conduct of the review and did the report justify any significant deviations from the protocol? (3) Did the review authors explain their selection of the study designs for inclusion in the review? (4) Did the review authors use a comprehensive literature search strategy? (5) Did the review authors perform study selection in duplicate? (6) Did the review authors perform data extraction in duplicate? (7) Did the review authors provide a list of excluded studies and justify the exclusions? (8) Did the review authors describe the included studies in adequate detail? (9) Did the review authors use a satisfactory technique for assessing the risk of bias (RoB) in individual studies that were included in the review? (10) Did the review authors report on the sources of funding for the studies included in the review? (11) If meta-analysis was justified, did the review authors use appropriate methods for the statistical combination of results? (12) If a meta-analysis was performed, did the review authors assess the potential impact of RoB in individual studies on the results of the meta-analysis or other evidence synthesis? (13) Did the review authors account for RoB in individual studies when interpreting and discussing the results of the review? (14) Did the review authors provide a satisfactory explanation for, and discussion of, any heterogeneity observed in the results of the review? (15) If they performed quantitative synthesis, did the review authors carry out an adequate investigation of publication bias (small study bias) and discuss its likely impact on the results of the review? (16) Did the review authors report any potential sources of conflicts of interest, including any funding they received for conducting the review? Scored as YES or NO. Partial Yes was considered as a point.	16	12	Denzer N et al. (3), Mahamat-Saleh Y et al. (5)

We chronologically arranged the studies according to the year they were published. We included the type of study, the size, the outcome, as well as the conclusion of each study, as shown in Table 3. Because few reports were found, the investigators decided to include one systematic review and one meta-analysis study. The meta-analysis study [5] investigated the risk of 25-hydroxyvitamin D levels, vitamin D intake, and skin cancer ( both melanoma and non-melanoma skin cancers), and we included four studies [12-14,15,16] mentioned in the meta-analysis that are relevant to basal cell carcinoma. 

**Table 3:** Information on the 10 studies that were included in the systematic review, arranged chronologically according to the published year. BCC: basal cell carcinoma, NMSC: non-melanoma skin cancer, 25(OH)D: 25-hydroxy vitamin D

Author	Year of study	Study design	Size	Outcome	Conclusion
Mahamat-Saleh Y et.al. [5]	2020	Meta-analysis	Four studies are included [12,14,15,16]	Every 30 nmol/L increment in 25(OH)D was associated with a 41% increase in BCC.	A higher relative risk was discovered at approximately 60 nmol/L of 25(OH) D, with a weaker association found above this level.
Vornicescu C et.al. [2]	2020	Case-control study	101 Romanians		In both controls and patients, vitamin D levels were deficient more than expected.
Ince B et al. [6]	2019	Case-control study	496 participants		Putting patients with BCC on vitamin D3 replacement therapy reduced the recurrence of BCC.
Park SM et al. [12]	2016	Cohort study	63,760 women and 41,530 men		Higher intakes of vitamin D from food and supplements are associated with an increased risk of BCC.
Denzer N et.al. [3]	2013	Systematic review	563 NMSC		There is an association between ApaI polymorphism and NMSC
Liang G et al. [15]	2012	Cohort study	4641 women		Positive correlation with NMSC risk, particularly with BCC and vitamin D.
Eide MJ et al. [14]	2011	Cohort study	3223 white men		Higher vitamin D levels (greater than 15 ng/mL) were positively correlated with developing NMSC
Lesiak A et al. [19]	2011	Case-control study	142 patients with BCC and 142 controls		The median serum level of 25(OH)D in the control group was significantly higher than in the BCC patients.
Tang JY. et.al. [4]	2011	Randomized controlled trial	36,282 postmenopausal women	Total of 1,655 NMSC cases in the placebo and 1,683 NMSC cases in the active CaD group	No difference between the groups.
Tang JY et al [16]	2010	Prospective cohort study	1,608 men		Decreased risk of NMSC in older Caucasian men who have higher serum 25(OH)D levels.

Comparison and Outcomes

**Figure 2:**
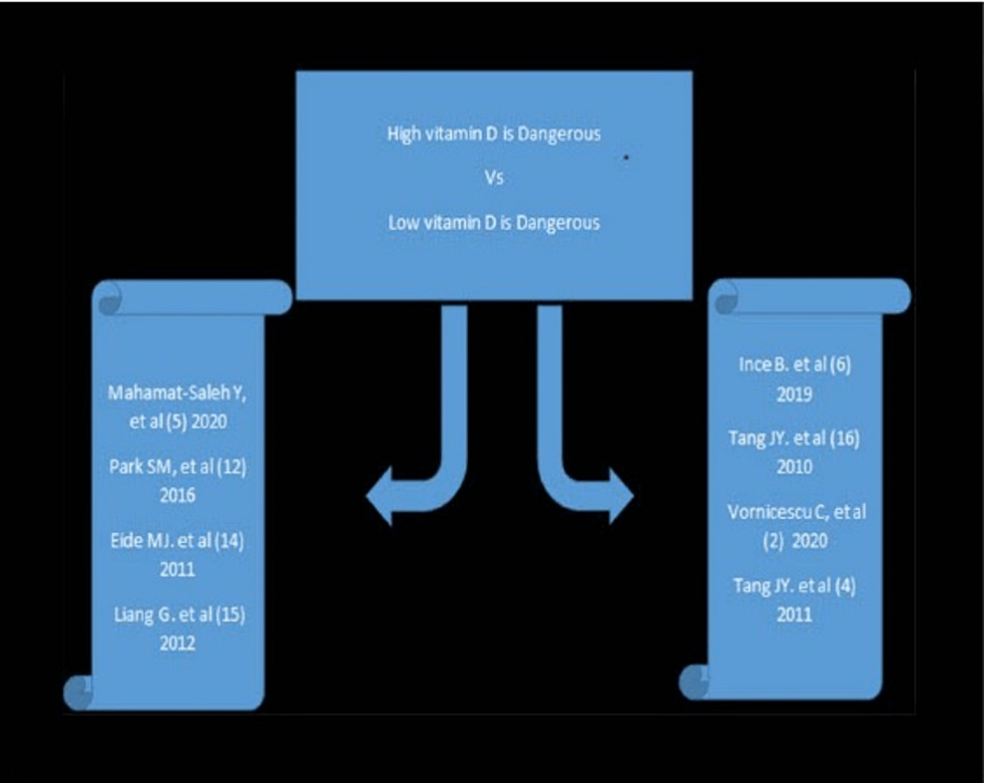
A flowchart depicting the studies that support the association of BCC with high serum vitamin D levels (four studies on the left) and the studies that support the association of BCC with low levels of vitamin D (four studies on the right).

The outcomes were divided into two groups; the first group supported the association between BCC and high levels of serum vitamin D, while the second group linked the association of low vitamin D serum levels with BCC. These studies are mentioned in Figure 2.

## Review

Discussion 

Vitamin D Synthesis and Benefits 

The metabolism of vitamin D is known to start with 7-dehydrocholesterol interacting with UVB to form vitamin D3, which then undergoes a hydroxylation reaction in the liver to form 25-hydroxyvitamin D3, to be converted to 1-alpha, 25-dihydroxy vitamin D3 in the kidneys, the metabolic active form. The only organ in our body that is capable of producing all components of vitamin D3 is our skin. The skin is capable of doing a fantastic job in response to exposure to ultraviolet B (UVB, 290-320 nm). Based on some studies, the skin epidermis has hydroxylases that can actively transform vitamin D3 to 25-hydroxyvitamin D3 without the involvement of the liver [6]. 

Vitamin D plays numerous protective roles in bone health [17], as well as in the prevention of certain diseases and cancers via signaling through vitamin D receptors (VDR). Interestingly, it has been observed in studies that women with high UVB exposure are 50% less likely to develop breast cancer in comparison with women with low UVB exposure [6,8,11]. In another study, prostate cancer incidence was 50% lower in men with high UVB exposure [9]. Lappe et al. reported that vitamin D3 and calcium supplementation significantly reduced all cancer risks in postmenopausal women. It was shown in a case-control study that the risk of nonmelanoma skin cancer (NMSC) development was lower in patients with high circulating pre-vitamin D metabolites, as well as reducing metastasis and increasing survival. Some of these studies showed that vitamin D exerts this desired inhibitory effect when binding to their specific receptors [10,13]. There are a huge number of vitamin D supplements sold every year. The sales increased to USD 963 million in 2017. That's almost a ninefold increase over the previous decade [11]. 

Approximately 80-90% of vitamin D is synthesized in our skin from sun exposure, particularly exposure to UV radiation (wavelength range between 280 and 320 nm), and the rest is from diet and supplement intake. Previous studies suggested that high plasma/serum 25(OH)D levels protect against a variety of chronic diseases, including cancer risk [5,8]. Several animal and human studies have demonstrated that vitamin D status may influence some cancers, such as colon, stomach, kidney, as well as skin, through down-regulation of cell growth [5] and modulation of the immune system [7]. As UV radiation is known to be a major skin carcinogen, especially for the Caucasian population, because it damages DNA in the skin cells [5], we are interested in studying the association between serum levels of vitamin D and BCC. There is a strong debate among scientific and public communities about the balance between the potential advantages and disadvantages of both UV-induced vitamin D production and vitamin D supplement intake and skin cancer prevention. 

VDR Polymorphism and Vitamin D 

Photochemical changes in the skin are introduced by UV-B radiation, leading to skin cancer [3], which is believed to be induced by a polymorphism in the VDR and MTHFR genes [7,19]. The VDR belongs to the superfamily of transacting transcriptional regulatory factors, including the steroid and thyroid hormone receptors as well as the retinoid-X receptors and retinoic acid receptors. Its gene is located on chromosome 12q12-14 [3]. VDR is expressed in many cells, including epidermal basal keratinocytes [2,19]. 1,25(OH)2D3 exerts its action by binding to VDR, which then translocates to the nucleus where it regulates the expression of VDR-responsive genes. Some of these genes are involved in cell differentiation and proliferation suppression [19]. The VDR gene is highly polymorphic as it has more than 618 variants. VDR genotypes differ tremendously by ethnicity [7]. Most of the polymorphisms that have been studied in association with skin cancer risk are FokI (rs2228570), ApaI (rs7975232), BsmI (rs1544410), and TaqI (rs731236) [19]. Studies show that the f allele of FokI occurs less frequently in Africans when compared to Caucasians and Asians [7]. 

The FokI polymorphism corresponds to a C/T substitution in exon four, which leads to a new translation initiation site and a longer VDR protein that is less transcriptionally active [19]. This is believed to be due to the presence of an F allele at FokI, which leads to the production of a longer protein (three more amino acids) than that produced by the f allele, i.e., the F allele exerts less transcriptional activity than the shorter protein produced by the F allele [7]. The other three are single nucleotide polymorphisms; ApaI and BsmI located in intron 10, and TaqI in extron 11, which don't change the amino acid sequence but they may decrease 1,25-dihydroxy vitamin D intracellularly or the VDR mRNA stability [19].

In a systematic review published in 2011, they gathered information about the vitamin D endocrine system (VDES), linking VDR polymorphism's role in the pathogenesis and occurrence of malignant melanoma (MM) and nonmelanoma skin cancer (NMSC) (including both BCC and SCC). They studied the following single nucleotide polymorphism (SNPs): TaqI (three studies and two reviews), BsmI (two studies and three reviews), FokI (three studies and three reviews), ApaI (one study), A-1012G (three studies and one review), BglI (one study) and Cdx2 (one study and two reviews). They concluded that there was an association between ApaI polymorphism and NMSC [3].

In a comprehensive meta-analysis in 2014, they collected data regarding the VDR and its role in cancer risk. Among these studies, only seven studies discussed skin cancer risk [7]. These studies compared the ff allele gene vs FF allele and Ff vs FF alleles, supporting the hypothesis that the f allele can interact with other factors like skin type and the presence of moles and nevi, and they concluded that the presence of the f allele was associated with a 24% significant increase in total skin cancer [7,18].

In addition, in 2011, they assessed the polymorphism of VDR genes and the risk of BCC in a case-control study in which they enrolled 142 patients with BCC and 142 controls, collected blood samples for DNA genotyping from all participants, and measured serum 25(OH)D by RIA in 79 BCC patients and 46 controls. The polymorphisms were accessed by polymerase chain reaction-restriction fragment length polymorphism (PCR-RFLP) and the VDR proteins were investigated by immunoblotting [19]. They discovered that: 1. the TT genotype in the Fokl VDR polymorphism increased the risk of BCC tenfold; 2. the expression of VDR proteins was significantly higher in BCC patients than in control skin, and 3. the median serum level of 25(OH)D was significantly higher in the control group compared to the patients with BCC [19].

Analyzing and discussing these data, we suggest the possibility of VDR polymorphism causing vitamin D levels to go down. Thus, when measuring the serum levels, they were found to be low, meaning that both low vitamin D levels and BCC are consequences of the polymorphism and that the low level of vitamin D was not the cause of BCC.

Vitamin D Serum Levels Concerning BCC Occurrence and Recurrence and Dose-Response 

According to a linear dose-response meta-analysis in 2020 conducted by Yahya Mahamat-Saleh et al, every 30 nmol/L increment in 25(OH)D was associated with a 41% increase in BCC, while according to a non-linear dose-response meta-analysis the association was significant for keratinocyte cancers (KC), with a higher relative risk found at a level of approximately 60 nmol/L of 25(OH) D, and a weaker association beyond this level [5]. 

A study conducted in 2016, where they sorted participants according to age, included 63,760 women and 41,530 men [12]. The medians of total vitamin D intake for extreme quintiles were 124.8 and 638.2 IU/d for women, and 156.0 and 775.3 IU/d for men. They noticed that older participants had higher vitamin D intake and higher levels of physical activity, as well as higher discipline in applying sunscreen. They followed up with the participants for 24-26 years, where they documented 20,840 incidents of BCC, 2,329 incidents of squamous cell carcinoma (SCC), and 1,320 melanomas. In that study, they documented the increased risk of BCC with higher intakes of vitamin D from food and supplements. Interestingly, participants who daily took vitamin D supplements ≥ 400 IU/d had a higher risk of BCC than those who didn't. Among the food items containing a high dose of vitamin D3, dietary intakes of total fish, low-fat/skim milk, and cereal showed significant positive associations with the risk of BCC [12].

In a study conducted in 2011, 3223 white health maintenance organization patients who sought medical advice for osteoporosis and bone issues from 1997 to 2001 were investigated for their vitamin D levels at their initial appointment. A deficient level of vitamin D was less than 15 ng/mL, whereas a normal, i.e., a sufficient level was equal to or more than 30 ng/mL. Among those, 240 patients were found to have NMSC: 49 had SCC, and 163 had BCC. They found that higher vitamin D levels (greater than 15 ng/mL) were positively correlated with developing NMSC. This correlation was positive though non-significant in less UV-exposed areas [14]. 

In a prospective study conducted in 2012, 4641 women from the Nurses' Health Study (NHS) and the NHS II were examined for NMSC (BCC and SCC). Five hundred and ten incidents of BCC and 75 incidents of SCC were found. Measuring plasma vitamin D levels, it was noticed that there is a positive association with the risk of NMSC, especially with BCC [15].

Figure 2 depicts a flowchart emphasizing the studies that support the association of BCC with high serum vitamin D levels (four studies on the left) and the studies that support the association of BCC with low levels of vitamin D (four studies on the right).

On the contrary, a study conducted in 2019 investigated the relationship between BCC and 25-OH vitamin D deficiency. A total of 496 patients enrolled in the study that took place in three stages, where the mean age was 69, and they were followed up for 24 to 36 months. Table 4 shows the participants in each stage. In the third stage, 50,000 IU of oral vitamin D3 was given per week for six weeks as a loading dose, totaling 300,000 IU, to patients who were diagnosed with BCC between February 2015 and January 2017 and who had a negative surgical margin of > 0.5 cm. 

**Table 4:** Participants in each of the three stages and the outcome after 50,000 IU of oral vitamin D3 was given per week for six weeks as a loading dose.

Stage	Men	Women	Serum 25-OH Vit D level (mean)	Recurrence
Stage 1	141	93	12.2 (male, 12.4; female, 11.9) ng/mL.	
Stage 2	60	54	10.1 (male, 10.2; female, 10.1) ng/mL.	9.64% (male, 10%; female, 9.26%)
Stage 3	84	59	11.8 (male, 11.7; female, 12) ng/mL	3.49% after replacement therapy

The mean serum 25-OH vitamin D3 level after replacement therapy was 40.1 (range: 28.9-65.2; male-42.3; female-37) ng/mL, and recurrence was 3.49% (male-3.57%; female-3.38%) of these patients.

In this study, they concluded that there is a statistically significant relationship between both primary and recurrent BCC and low serum 25-OH vitamin D3 levels. Additionally, putting patients with BCC on vitamin D3 replacement therapy reduced the recurrence. As a result, this study suggested maintaining a level of >25 ng/mL of 25-OH vitamin D3 in those diagnosed with BCC, as this serum level can significantly reduce recurrence. And because of the paradoxical effect of UVB, patients were advised to avoid sunlight at noon and avoid exposing the dangerous previously affected areas, but they may expose other BCC-free areas during daylight for 10-15 minutes, or they may consider taking vitamin D3 supplements to maintain the 25 ng/mL of Vit D [6].

In 2010, a prospective cohort study was conducted on 5,995 community-dwelling men (65 years and older) from March 2000 to April 2002. They were participating in the Osteoporotic Fractures in Men Study (MrOS), a prospective cohort study of risk factors for fracture [17]. They measured both 25(OH)D2 and 25(OH)D3 in randomly selected 1,608 participants, and they concluded that there is a decreased risk of NMSC in older Caucasian men who have higher serum 25(OH)D levels [16].

In 2020, an observational analytical transversal case-control study was published, where they investigated 101 Romanian participants and divided them into four sub-groups: 1. with one BCC group; 2. with more than one BCC group; 3. recurrent BCC group; and 4. with both multiple and recurrent. Patients with one or more were 52, and controls with no history of skin cancer were 59. They measured serum 25-OH-D levels. In this study, they found that vitamin D levels are deficient more than expected in both controls and patients due to skin diseases and that there is a lack of awareness in general in the population [2].

In a randomized control trial held in 2011, 36,282 postmenopausal women (age 50 to 79 years) were enrolled in the Women's Health Initiative (WHI) calcium/vitamin D clinical trial and were randomly provided 1,000 mg of elemental calcium plus 400 IU of vitamin D3 (CaD) daily or placebo for a mean follow-up period of 7.0 years [4]. There was no difference between both groups, with a total of 1,655 NMSC cases in the placebo group and 1,683 NMSC cases in the active CaD group. They noticed that CaD supplementation reduced the incidence of melanoma in women with a history of NMSC, assuming the association of either vitamin D or calcium or their combination in decreasing the risk of melanoma in the high-risk group. Still, they don't recommend using these supplements in these doses in older women for reducing NMSC [4].

Limitations

This review has several limitations. As much as we tried to include basal cell carcinoma studies only in our review, we still had two of our studies mention their cases as non-melanoma skin cancers (NMSC), i.e., BCC and SCC [3,7]. Also, few studies have addressed the relationship between vitamin D serum level, VDR polymorphism, and BCC, which may affect the overall conclusion as the investigators included one meta-analysis that mentioned four of the included studies in the systematic review.

## Conclusions

There are a lot of debates and controversial studies when it comes to the risks and benefits of increasing vitamin D intake. However, several studies suggest the protective role of vitamin D when it comes to developing BCC. Many factors play a role in the development of BCC; some of them are controllable, like sun exposure, and others are not, like ethnicity and genetic factors. As mentioned in the biggest meta-analysis we included, they suggest keeping the serum level of vitamin D at a level below approximately 60 nmol/L of 25(OH) D as there was a weaker association with BCC. Another study supported that a level of vitamin D greater than 30 ng/mL was associated with BCC. We concluded that keeping vitamin D levels at or below 30 ng/mL is considered safe. Having levels that are so low, i.e., below 15 ng/mL, would add to the risk of its progression as supported by the other team, so we suggest staying above 15 ng/mL but below 30 ng/mL, considering it to be a balanced level.

After studying these reports, raising awareness of the potential risks that high vitamin D serum levels may hold for increasing BCC incidence should be encouraged. More studies should address the relationship between VDR polymorphism, BCC, and vitamin D serum levels. Studies that support that low vitamin D levels are associated with increasing BCC may further investigate the relationship between VDR polymorphism and BCC as well, to identify the most possible cause of BCC. For the studies that support the association of BCC with high vitamin D levels, studying the presence or absence of VDR polymorphism is a means to explore the true effect of the polymorphism on vitamin D serum levels. Few studies were done on BCC and its relationship with vitamin D serum levels, encouraging more studies to be done specifically on BCC rather than concluding it as an NMSC would be very beneficial.
